# Carbon dioxide narcosis in the terminal stage of hemodialysis therapy: A case report with the possible pathophysiologies and the treatment methods

**DOI:** 10.1002/ccr3.4053

**Published:** 2021-03-13

**Authors:** Akira Takahashi

**Affiliations:** ^1^ Tesseikai Neurosurgical Hospital Dialysis Center Shijonawate Japan

**Keywords:** carbon dioxide (CO_2_) narcosis, carbonic anhydrase, carnitine, coenzyme Q10 (CoQ10), hypozincemia

## Abstract

The cause and treatment of carbon dioxide narcosis in the terminal stage of hemodialysis have not been fully discussed. As we have experienced the case of complete recovery, we report the possible pathophysiologies and the treatment methods.

## Graphical abstract

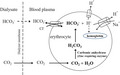

## 1 INTRODUCTION

We report a case in which an 85‐year‐old woman who was hospitalized for end‐of life care developed CO_2_ narcosis during hemodialysis therapy but was able to recover enough to walk alone. One of the causes is hypercapnia due to insufficient carbonic anhydrase of erythrocytes, which is a zinc‐requiring enzyme.

Chronic hypercapnia is often observed in the terminal stage of dialysis in elderly patients, eventually causing carbon dioxide (CO_2_) narcosis and often discontinuing spontaneous breathing. The cause and treatment of CO_2_ narcosis in the terminal stage of hemodialysis have not been fully discussed. We experienced a case in which a patient who was hospitalized for end‐of life care developed respiratory arrest owing to CO_2_ narcosis during hemodialysis therapy but was able to recover enough to walk on her own. We report the possible pathophysiologies causing CO_2_ narcosis during hemodialysis therapy and the treatment methods.

## 2 CASE REPORT

An 85‐year‐old woman with chronic hypoproteinemia, heart failure, and pleural effusion was referred to our hospital for maintenance hemodialysis treatment and end‐of‐life care in the terminal stage of hemodialysis. She was diagnosed with end‐stage renal failure owing to nephrosclerosis 2 years and 4 months previously (at 81 years of age) and selected hemodialysis as a renal replacement therapy. She was treated at a local dialysis clinic three times a week for maintenance hemodialysis. She had a medical history of an operation for cervical spinal canal stenosis 15 years earlier and a transient cerebral ischemic attack 13 years earlier. There was no special mention of either of these conditions in her family history, and she had no allergies. She was hospitalized at another hospital for aspiration pneumonia because of difficulty swallowing 3 months earlier, and pleural effusion was noted at that time. Later, sarcopenia and malnutrition worsened, and 2 weeks before admission, she experienced apneic attacks twice during maintenance hemodialysis therapy at the clinic. She was gradually losing her ability to walk and required wheelchair assistance. The dialysis conditions were a duration of 4 hours, dialyzer: FB‐150Uβ (NIPRO Co., Ltd.), and blood flow rate: 150 mL/min. The dialysate was Carbostar^®^ (Ajinomoto Pharmaceuticals Co., Ltd.), which contains 35 mEq/L of bicarbonate (HCO_3_−). She was taking lansoprazole 15 mg, clopidogrel 75 mg, azilsartan 20 mg, sodium ferrous citrate 50 mg, pregabalin 50 mg, fexofenadine hydrochloride 60 mg, and arotinolol 5 mg.

On examination at admission, her body temperature was 36.5°C, blood pressure: 144/60 mm Hg, heart rate: 65 beats per minute, respiratory rate: 17 breaths per minute, and oxygen saturation: 87% while breathing room air. Her body mass index (weight in kg divided by the square of the height in meters) was 14.9 kg/m^2^, and her dry weight was 30.0 kg. She had a slightly increased work of breathing and diffuse coarse crackles in both lung fields. The remainder of the physical examination was normal. Arterial blood gas analysis revealed hypercapnia, with a pH of 7.250, partial pressure of CO_2_ (PCO_2_): 63.1 mm Hg, partial pressure of oxygen (PO_2_): 56.1 mm Hg, HCO_3_−: 27.1 mmoL/L, base excess (BE): −1.0 mmoL/L, and total carbon dioxide (tCO_2_): 29.0 mmoL/L (Table 1). Her red blood cell count (RBC) was 322 × 10^4^/μL, hemoglobin (Hb): 10.0 g/dL, hematocrit (Hct): 32.5%, mean corpuscular volume (MCV): 100.9 fL, mean corpuscular hemoglobin (MCH): 31.1 pg, platelet count (PLT): 17.4 × 10^4^/μL, iron (Fe): 22 μg/dL, total iron‐binding capacity (TIBC): 226 μg/dL, transferrin saturation (TSAT): 9.73, and ferritin: 55 ng/mL. The serum zinc concentration was 37 μg/dL (reference range: 80‐130 μg/dL), and the copper concentration was 131 μg/dL (reference range: 71‐132 μg/dL). The free carnitine concentration was 15.7 μmoL/L (reference range: 36.0‐74.0 μmoL/L), acylcarnitine: 5.0 μmoL/L (reference range: 6.0‐23.0 μmoL/L), and the acylcarnitine‐to‐free carnitine (AC/FC) ratio was 0.318. A chest radiograph obtained on admission showed bilateral pleural effusion with patchy opacities in the right lower lung fields. Because we considered that the hypoventilation, which is a cause of hypercapnia, was caused by alkalosis following the prolonged hemodialysis of 4 hours despite her advanced age, light weight, and poor nutritional status, we decreased the dialysis time to 3 hours. The dialyzer was an APS‐15SA (Asahi Kasei Medical Co., Ltd., Tokyo, Japan), and blood flow rate was 150 mL/min, which was the same as before hospitalization. We changed the dialysate from Carbostar (HCO_3_^−^: 35 mEq/L) to Kindaly 3E (Fuso Pharmaceutical Industries, Osaka, Japan) (HCO_3_^−^: 25 mEq/L), to obtain a lower bicarbonate concentration. We also began administering carnitine, CoQ10, and water‐soluble vitamins. Because the respiratory muscle weakness, which was a cause of the worsening hypoventilation, was caused by insufficient ATP supply secondary to malnutrition and age‐related sarcopenia, we administered an intravenous injection of levocarnitine. Concurrently, we began oral administration of ubidecarenone (CoQ10) and intravenous infusions of water‐soluble multivitamins. Despite these measures, CO_2_ narcosis recurred during the second dialysis treatment after hospitalization (Table 1), followed by ceasing spontaneous breathing, and we immediately initiated noninvasive positive pressure ventilation (NPPV). The preliminary diagnosis was CO_2_ narcosis, which was thought to have occurred because of a lack of ventilation from respiratory muscle weakness and hypofunctioning carbonic anhydrase in red blood cells secondary to hypozincemia. We initiated zinc supplementation with oral administration of zinc acetate hydrate 50 mg and intravenous infusion of multiple trace elements (Fe, Mn, Zn, Cu, I) in the hyperalimentation fluid. Sodium ferrous citrate, which may compete for zinc absorption, was discontinued. Pregabalin, which may suppress respiratory muscles, was also discontinued. She required NPPV therapy for 19 hours, but then recovered enough to regain spontaneous breathing. Arterial blood gas analysis also returned to manageable ranges without NPPV (Table 1) as follows: pH: 7.315, PCO_2_: 52.0 mm Hg, PO_2_: 72.2 mm Hg, HCO_3_^−^: 25.9 mmoL/L, BE: −0.7 mmoL/L, and tCO_2_: 27.5 mmoL/L. Apnea did not recur for 2 weeks after the second day of hospitalization. On the fourteenth day of admission, serum zinc concentration had improved to 122 μg/dL, and the patient's respiratory condition during dialysis was stable with the following arterial blood gas analysis results: pH: 7.351, PCO_2_: 42.5 mm Hg, PO_2_: 81.2 mm Hg, HCO_3_^−^: 23.0 mmoL/L, BE − 2.5 mmoL/L, and tCO_2_: 24.3 mmoL/L (Table 1). On the sixteenth day of hospitalization, she was able to walk on her own and was discharged. Currently, 10 months have passed since she was discharged, and she continues maintenance dialysis at our outpatient department. Her dry weight has increased to 38.0 kg, with a body mass index of 18.8 kg/m^2^. Consent was obtained from the patient for submission of this case report.

**TABLE 1:** Arterial blood gas analysis measured on admission and at three later time points

	pH	PCO_2_ (mm Hg)	PO_2_ (mm Hg)	HCO_3_^−^ (mmoL/L)	BE (mmoL/L)	tCO_2_ (mmoL/L)
On admission	7.250	63.1	56.1	27.1	−1.0	29.0
When CO_2_ narcosis occurred	7.147	74.2	52.2	25.1	−4.7	27.4
1 d after CO_2_ narcosis	7.315	52.0	72.2	25.9	−0.7	27.5
2 wk after CO_2_ narcosis	7.351	42.5	81.2	23.0	−2.5	24.3

Abbreviation: BE, base excess; HCO_3_^−^, bicarbonate; PCO_2_, partial pressure of carbon dioxide; PO_2_, partial pressure of oxygen; tCO_2_, total carbon dioxide.

## 3 DISCUSSION

**FIGURE 1:**
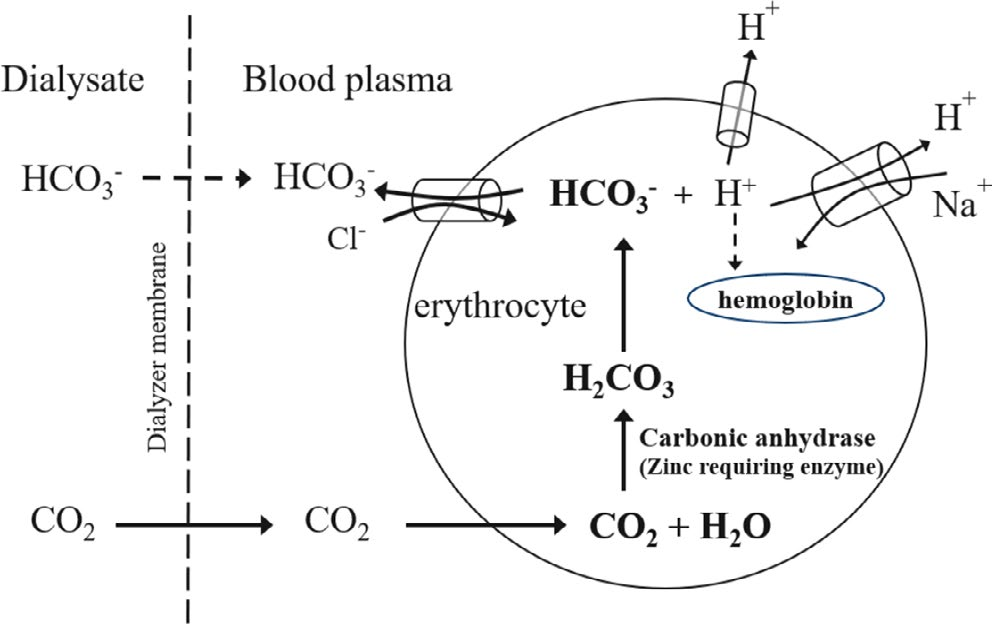
Movement of carbon dioxide in the dialyzer when using sodium bicarbonate‐containing dialysate. CO_2_, carbon dioxide; HCO_3_^−^, bicarbonate; Cl^−,^ chloride; H^+^, hydrogen; Na^+^, sodium; H_2_O, water; H_2_CO_3_, carbonic acid

The pathophysiology of alveolar hypoventilation causing hypercapnia may involve decreased central drive, drugs (sedatives), disuse atrophy, lung and airway disease, and other causes.^1^ In dialysis patients, decreased central drive may result from hyperalkalosis secondary to excessive dialysis.^2^ Ogata et al reported a method to prevent carbon dioxide narcosis by lowering the bicarbonate concentration of the dialysate.^3^ A method of using acetate‐free biofiltration as a CO_2_‐free treatment for hemodialysis patients with hypercapnia has also been reported.^4^ In our case, we lowered the amount of dialysis and total bicarbonate concentration by reducing the dialysis time from 4‐3 hours and by changing the dialysate to one with a lower HCO_3_^−^ concentration. Analgesics, which possibly suppressed the respiratory center, were also thought to have caused hypoventilation. Therefore, pregabalin, which may suppress respiratory muscles,^5^ was discontinued. Another cause of ventilatory impairment that leads to chronic hypercapnia is disuse atrophy and respiratory muscle weakness secondary to low ATP.^6^ In the terminal stage of dialysis, older adult patients often have protein energy wasting (PEW), which is caused by chronic inflammation, catabolism, and malnutrition. PEW is also considered to be related to respiratory muscle weakness.^3^ Despite decreasing the dialysis time and changing the dialysate, CO_2_ narcosis recurred, in our patient. We believe that hypozincemia, which was observed at admission, may have exacerbated the CO_2_ narcosis by inducing hypercapnia secondary to carbonic anhydrase dysfunction in erythrocytes, in addition to the insufficient ventilation because of respiratory muscle weakness. Although CO_2_ narcosis may be common after administering oxygen to hypercapnic patients, CO_2_ narcosis during hemodialysis therapy can occur without oxygenation, as in our case. Sodium bicarbonate dialysates (7% CO_2_ dialysate), such as Kindaly^®^ 3E, supply CO_2_ instead of HCO_3_^−^ from the dialysate to the blood through the dialyzer membrane. PCO_2_ in blood and dialysate are measured before and after the dialyzer. For example, after 2 hours, the PCO_2_ of dialysate is as high as 52.6 ± 2.4 mm Hg, so after passing through the dialyzer, the blood PCO_2_ increased significantly from 33.6 ± 4.4 mm Hg to 42.8 ± 2.6 mm Hg (*P* <.001), but HCO_3_^−^ did not change significantly from 24.1 ± 1.5 mm Hg to 24.5 ± 1.3 mm Hg. In this way, it is not HCO_3_^−^, but carbon dioxide that is supplied to the blood from the dialysate.^7^ CO_2_ passes easily through the erythrocyte phospholipid bilayer membrane and is converted to HCO_3_^−^ 5000‐15 000 times faster by the action of carbonic anhydrase within the erythrocytes. Carbonic anhydrase is a zinc‐requiring enzyme.^8^ HCO_3_^−^ is exchanged for chloride (Cl^−^) and enters the plasma. In fact, as blood passes through the dialyzer, serum chloride ion concentrations fall below that of the dialysate.^7^ (Figure 1) HCO_3_^−^ is not supplied directly from the dialysate, but is produced in erythrocytes and enters the plasma by the effects of carbonic anhydrase, which is abundant in erythrocytes. Therefore, zinc is essential for alkalization in hemodialysis therapy. With zinc deficiency, CO_2_ cannot be processed, and arterial CO_2_ partial pressure increases, which causes CO_2_ narcosis. Spontaneous respiration may cease during hemodialysis therapy, as a result.

Care should be exercised in older adult dialysis patients with malnutrition because hypercapnia can occur secondary to ventilatory impairment from weakened respiratory muscles as well as by carbonic anhydrase dysfunction secondary to zinc deficiency. Decreased plasma zinc may develop in patients with end‐stage renal failure undergoing hemodialysis because of redistribution, rather than systemic deficiency.^9^ In renal failure, plasma zinc concentrations decrease, but the zinc concentration in erythrocytes increases.^10^ This abnormal distribution is mainly caused by carbonic anhydrase in erythrocytes being a zinc‐requiring enzyme.^8^ Carbonic anhydrase and zinc concentrations in erythrocytes are significantly increased in chronic hemodialysis patients compared with healthy adults.^11^

To restore the strength of weakened respiratory muscles, which are responsible for poor ventilation, it is necessary to restore the energy circuit of the ATP supply. Carnitine plays a role in transporting fatty acids to mitochondria, but carnitine is involved only up to acyl‐CoA in the ATP cycle. When acyl‐CoA is transported to the beta‐oxidation system and is further processed in the tricarboxylic acid (TCA) cycle, water‐soluble vitamins are required, and these are removed during dialysis therapy. Subsequent electron transport systems require CoQ10, which is decreased by aging,^12^ as well as magnesium and iron. In the nutritional management of hemodialysis patients developing CO_2_ narcosis, supplementing all of these factors is necessary to provide a sufficient supply of ATP to the respiratory muscles.

One cause of zinc deficiency is lack of intake.^13^ Most dialysis patients are older adults and have low dietary intake. Furthermore, these patients often have a high prevalence of polypharmacy.^14^ Absorption disturbance because of the intake of zinc absorption inhibitors is also a cited cause of hypozincemia,^15^ and dietary fiber, oxalic acid, and phytic acid in beans and other plants inhibit zinc absorption.^16^ Calcium and iron also inhibit zinc absorption.^17^ In one study, serum carbonic anhydrase concentrations did not change after iron supplementation, but the concentrations of zinc in erythrocytes decreased in chronic kidney disease (CKD) patients.^18^ Furthermore, zinc absorption is inhibited when taking medications containing calcium carbonate or iron to treat hyperphosphatemia. Our patient was treated with sodium ferrous citrate to treat iron deficiency anemia. Magnesium also competes with zinc during absorption,^19^ and hypozincemia may also be caused by medications that chelate zinc. Zinc deficiency causes taste disorders,^20^ but many drugs have this effect, and most are zinc chelating agents.^21^ In addition, prescriptions for proton‐pump inhibitors and histamine‐2 receptor antagonists are increasing in dialysis patients^22^; however, caution is necessary because the long‐term use of drugs that suppress gastric acid secretion decreases the absorption of zinc and magnesium.^23^ Excessive loss also causes zinc deficiency,^24^ and hemodialysis removes not only uremic toxins, but also zinc, water‐soluble vitamins, and carnitine.

In conclusion, to prevent CO_2_ narcosis in patients in the terminal stage of hemodialysis therapy, if hypozincemia is present, zinc supplementation to improve the function of erythrocyte carbonic anhydrase may increase CO_2_ processing capacity. Additionally, carnitine, COQ10, and water‐soluble vitamins are required to provide sufficient ATP to the weakened respiratory muscles. To decrease respiratory center suppression and increase ventilation capacity, it is also necessary to modify the dialysis volume and dialysate composition.

## CONFLICT OF INTEREST

There were no potential conflicts of interest noted.

## AUTHOR CONTRIBUTIONS

AT: involved in research idea, study design, data acquisition, and data analysis/interpretation. AT: takes responsibility that this study has been reported honestly, accurately, and transparently and accepts accountability for the overall work by ensuring that questions pertaining to the accuracy or integrity of any portion of the work are appropriately investigated and resolved.

## ETHICAL APPROVAL

Informed consent was obtained from the patient for being included in this case report.

## Data Availability

The data that support the findings of this study are available on request from the corresponding author, AT The data are not publicly available due to the containing information that could compromise the privacy of research participant.
